# Use of social network analysis methods to study professional advice and performance among healthcare providers: a systematic review

**DOI:** 10.1186/s13643-017-0597-1

**Published:** 2017-10-23

**Authors:** Kate Sabot, Deepthi Wickremasinghe, Karl Blanchet, Bilal Avan, Joanna Schellenberg

**Affiliations:** 1 The Centre for Maternal, Adolescent, Reproductive and Child Health (MARCH), London School of Hygiene & Tropical Medicine, Keppel Street, London, WC1E 7HT UK; 2 Department of Disease Control, Faculty of Infectious and Tropical Diseases, London School of Hygiene & Tropical Medicine, Keppel Street, London, WC1E 7HT UK; 3 Department of Global Health, Faculty of Public Health and Policy, London School of Hygiene & Tropical Medicine, London, UK

**Keywords:** Social network analysis, Health outcomes, Health system performance, Professional advice, Professional communication, Healthcare workers, Network analysis, Systematic review

## Abstract

**Background:**

Social network analysis quantifies and visualizes relationships between and among individuals or organizations. Applications in the health sector remain underutilized. This systematic review seeks to analyze what social network methods have been used to study professional communication and performance among healthcare providers.

**Methods:**

Ten databases were searched from 1990 through April 2016, yielding 5970 articles screened for inclusion by two independent reviewers who extracted data and critically appraised each study. Inclusion criteria were study of health care worker professional communication, network methods used, and patient outcomes measured. The search identified 10 systematic reviews. The final set of articles had their citations prospectively and retrospectively screened. We used narrative synthesis to summarize the findings.

**Results:**

The six articles meeting our inclusion criteria described unique health sectors: one at primary healthcare level and five at tertiary level; five conducted in the USA, one in Australia. Four studies looked at multidisciplinary healthcare workers, while two focused on nurses. Two studies used mixed methods, four quantitative methods only, and one involved an experimental design. Four administered network surveys, one coded observations, and one used an existing survey to extract network data. Density and centrality were the most common network metrics although one study did not calculate any network properties and only visualized the network. Four studies involved tests of significance, and two used modeling methods. Social network analysis software preferences were evenly split between ORA and UCINET. All articles meeting our criteria were published in the past 5 years, suggesting that this remains in clinical care a nascent but emergent research area. There was marked diversity across all six studies in terms of research questions, health sector area, patient outcomes, and network analysis methods.

**Conclusion:**

Network methods are underutilized for the purposes of understanding professional communication and performance among healthcare providers. The paucity of articles meeting our search criteria, lack of studies in middle- and low-income contexts, limited number in non-tertiary settings, and few longitudinal, experimental designs, or network interventions present clear research gaps.

**Systematic review registration:**

PROSPERO CRD42015019328

**Electronic supplementary material:**

The online version of this article (10.1186/s13643-017-0597-1) contains supplementary material, which is available to authorized users.

## Background

In 2015, the Millennium Development Goals (MDGs) expired after 15 years of galvanizing the global development community around health targets related to women, children, and HIV and AIDS. Their replacement, the Sustainable Development Goals (SDGs), broaden global focus beyond health [1]. As such, the health sector will need to explore new ways to influence provider practice and scale up best practices to meet the outstanding MDG targets and improve health outcomes. Understanding and harnessing the power of existing professional advice networks among healthcare providers could assist in influencing provider practice and improving health outcomes in low- and middle-income countries (LMIC). Social network analysis focuses on studying relationships between and among individuals (or organizations) who are connected by one or more ties of interdependency, such as love, friendship, kinship, trust, collaboration, or communication [2]. Social network analysis (SNA) can lend insight into defining, measuring, and understanding these professional communication networks and therefore designing effective network interventions to improve provider performance and ultimately, health outcomes [3, 4].

SNA is defined as a means of mapping and exposing channels of communication and information flow, collaboration, and disconnection between people [5]. SNA is both a theory and a methodology that has generated a body of empirical research [6, 7]. One of these theories is diffusion of innovations defined by Rogers as the “process by which an innovation is communicated through certain channels over time among members of social system” (p. 5) [8]. Rogers proposed that individuals go through several stages in deciding to “adopt” an innovation; a process influenced by the characteristics of innovations, specifically the complexity, triability, observability, and the relative advantage conferred by the innovation [8, 9]. Individual adoption of an innovation can be expressed as a normal distribution, segmenting individuals into categories of individual innovativeness: innovators, early adopters, early majority, late majority, and laggards [8].

Professional behavior change among healthcare providers is often referred to as knowledge translation or transfer. We hypothesize that certain network structures and the presence of network roles within networks of healthcare providers can facilitate diffusion of innovations, or knowledge translation and transfer, particularly where the issue is lack of provider information, and that may in turn change practices and improve patient outcomes. Admittedly, this is a simplification as relationships among healthcare providers are multiplex and friendship or trust networks rather than purely professional communications may be more influential in changing provider behavior when there is informational ambiguity [10]. As such, it is important to consider both formal and informal professional communication in an attempt to mitigate this concern.

While it is not possible for this paper to include a comprehensive overview of social network analysis study designs, data collection, and data analysis methods, the key concepts are highlighted and further explanations can be found elsewhere [2]. Social network analysis studies are defined primarily as either whole network, including all members of a group defined by a specified boundary or ego network studies, capturing the networks of select individuals within a network. Hybrid models can combine elements of both approaches. All networks are characterized by whether the network is “directed,” indicating the orientation of the relationship, for example if A influences B, then the tie would include an arrowhead at B or “undirected” where the relationship either exists or does not and none of the ties or lines have arrowheads. They are also either “valued” capturing the intensity of the relations on a scale or “unvalued,” whereby these relations are dichotomous. Network data can be captured through questionnaires, interviews, observations, existing records, diaries, or other methods [2]. Other data collection methods or ways of generating networks of providers include journal publication co-author lists, identifying patient-sharing among providers, attendance at conferences, and participation on social media forums, to name a few.

Social network analysis data analysis method options depend on how the data were collected and the research questions of interest. In SNA, visualizing data is both a means of presenting findings as well as a tool for generating additional findings. Quantification of network properties are subject to certain constraints as the unit analysis is a relationship between actors (individuals or organizations) rather than independent observations. Thus, SNA requires analytical tools that do not rely on independence of observations or relations [2]. Analysis can be at the actor, subgroup, or network level. Common subgroup structures are dyads, triads, clusters, cliques, components, and bridges [11]. Many network metrics can be calculated including degree, density, centrality, reachability, and distance. Some of these can be calculated at the network or actor level or both. Gesell et al. [12] recommend calculating isolates, degree, and reciprocity at the actor level, and at the network level. the presence of subgroups, density, centralization, transitivity, and cohesion as the metrics most likely to have effect on individual and group processes.

A 2012 systematic review of SNA applications in healthcare settings concluded that SNA’s potential has been unrealized in the health sector, particularly because virtually all identified studies were simple network descriptions rather than studies of network interventions [5]. This review had a definition of a healthcare setting that excluded community-based health workers and interventions, a limitation particularly relevant in LMIC and global health.

The present systematic review builds on the Chambers et al.’s [5] review in the following ways: broadening the definition of “healthcare settings” to be inclusive of community-based settings, expanding the databases and search terms, and updating the searches to include articles from 2011 to 2016. The focus of the review synthesis is substantively different looking specifically at SNA methods used to understand healthcare provider communication and performance. The primary research question this review sought to address is what SNA methods have been used to study professional communication and performance among healthcare providers? Secondary research questions included:

- Does professional communication improve health outcomes? What professional communication network properties are associated with health outcomes?
- What methods have been used for which types of research questions?
- What are the main limitations of the SNA methods?
- What is the quality of these studies?
- What is the quantity of SNA studies? What was the evolution over time?
- To what extent has this research taken place in low- and middle-income countries?
- To what extent has this research focused on community-based health providers?

## Methods

### Definitions

For any systematic review, it is critical to clarify our meaning when using terms that define a search strategy. For this review, we have operationally defined “healthcare providers,” “professional communication,” and “performance” as follows.

In this context, we defined “healthcare providers” as physicians, clinical officers, nurses, midwives, counselors, physician’s assistants, and others who provide health-related services to patients in formal medical environments. Additionally, community-based cadres such as community health workers, village health workers, traditional birth assistants, and others were also considered healthcare providers.

For our purposes, we defined “professional communication” as formal or informal professional advice-seeking or giving or discussion about hypothetical or actual work situations or patients. For example, studies exploring friendship networks of healthcare providers were not considered eligible, unless they also captured communication related to work situations or patient care and documented patient health outcomes.

We defined “performance” as a study including a patient health outcome. Studies that only considered “patient satisfaction” or healthcare provider “perceptions of performance” were not eligible for inclusion.

### Search strategy

The search strategy focuses on the intersection of SNA and diffusion of innovations, the term used in the SNA community most relevant for professional communication related to knowledge sharing and transfer. Since health policy and health systems research often use “knowledge translation or transfer” language, the search strategy also includes the intersection between those terms and SNA. As a methodologically focused review, this review will highlight the range of SNA methods applied.

To address the research questions, the systematic review focused on three concepts that are integral to the primary research question: (1) SNA, (2) diffusion of innovations, and (3) knowledge translation and transfer. The key terms for these concepts are shown in Additional file 1 and truncation search terms will be used to make the search inclusive.

#### Concept 1: social network analysis

The search strategy for the SNA concept was adapted from the Chambers et al.’s scoping systematic review of Social Network Analysis and healthcare settings [5]. This was particularly helpful guidance as a more recent SNA review; Cunningham et al. noted the challenge of “social network” yielding irrelevant social media or social support articles [13]. One of the changes from the Chambers et al. review was an expansion of the list of SNA software listed (from four: UCINET, NetDraw, Pajek, and KrackPlot to 56), which was guided by a chapter in the SAGE Handbook of Social Network Analysis [14]. Depending on the database, specific software packages (Blanche, InFlow, Jung, ORA, ORS, Pnet, Puck UNISoN, SNAP, and STRUCTURE) were excluded as they yielded thousands of off-topic articles. See Additional file 2 for a list of exclusions by database. None of these exclusions were the SNA packages included in the previous review and for the most part are not the most commonly used software packages for SNA. The one exception is ORA, a SNA software package that, for 6 of 10 databases, returned thousands of articles that used odd ratios in their analysis. However, as this review still yielded two studies that used ORA, we do not feel that this negatively impacted the search.

#### Concept 2: diffusion of innovations

The search strategy for the diffusion of innovations concept was influenced by the original search strategy used as a starting point for a meta-narrative on *Diffusion of Innovations in Health Service Organizations* [15]. However, the focus on health service organizations was seen as potentially too limiting. Therefore, terms related to health service organizations were not included to let the review capture a broader range of studies. “Diffusion of innovations” is a phrase that is relatively new to health systems research. Consequently, the review used a third concept to ensure all relevant studies were captured, which corresponds to diffusion of information: knowledge translation and transfer.

#### Concept 3: knowledge translation and transfer

Knowledge translation and transfer (KT) are terms describing a relatively new discipline, which does not have an agreed upon lexicon. A systematic study of KT terms used in 12 journals found inconsistent use of KT terms such that less than half of what the authors classified as “KT articles” used the presumed “KT terms” leading the authors to refer to the situation as a “tower of babel” [16]. The search strategy for this concept was developed by determining the common terms across six sources including four systematic reviews [17–20] and two articles on knowledge translation “KT” or “K*” terms [16, 21]. A comprehensive listing of all 253 K* terms can be found in Additional file 3. An initial search conducted using all the terms yielded over 6000 articles in MEDLINE, which led to a revision of the approach for this concept. Priority terms for inclusion in the search strategy were those that appeared in more than one source.

The search strategies were then developed looking at the intersection of concept 1 with concept 2 and the intersection of concept 1 with concept 3. They were then adapted to each of the databases included in the review, including mapping the above terms to MeSH terms. Detailed search strategies for each of the 10 databases are available upon request—an example is included in Additional file 4.

MEDLINE, EMBASE, PsychINFO, CINAHL, Global Health, Social Policy and Practice, Health Management Information Consortium, and Web of Science were searched. Gray literature was searched via Popline. The Cochrane Library was searched to identify other systematic reviews and relevant studies. Several websites were searched including International Network for Social Network Analysis, American Evaluation Association Social Network Analysis Technical Interest Group, and in the International Sunbelt Social Networks Conference proceedings archives.

Articles were downloaded into Endnote X5.0.01, a bibliographic software package and duplicates within and across databases were removed. All 5970 articles were then assessed for meeting study inclusion criteria through a three-stage review process. Two independent reviewers (KS and DW) screened titles, abstracts, and full-text articles; after each step, discrepancies were discussed and reconciled.

The 10 systematic reviews identified through the search strategies that addressed SNA had the articles they included screened for inclusion in this review [5, 22–29].

**Fig. 1:**
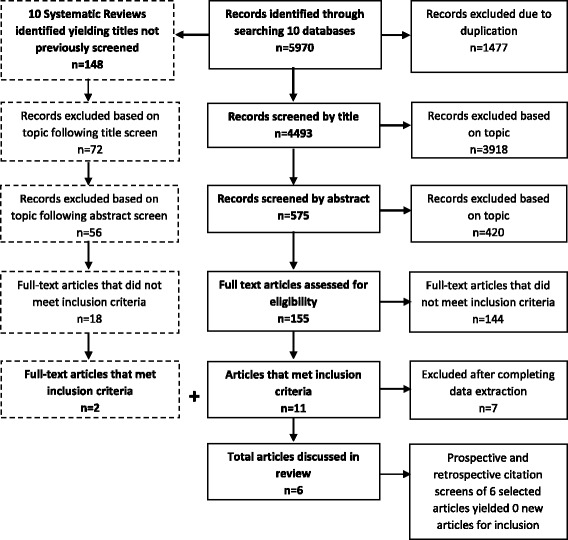
PRISMA flow chart

The search strategies were executed originally from 1990 to January–March 2015 and then updated in April 2016, capturing articles published since the original search. All systematic reviews identified had the articles they included screened. The final set of articles had their reference lists screened and SCOPUS was used to conduct a prospective citation search. All articles were subjected to our two independent reviewer, 3-stage screening process. The PRISMA flow chart (Fig.1) reflects the combination of the searches and screenings conducted in 2015 and updated in 2016.

The study protocol was registered with PROSPERO DOI: 10.15124/CRD42015019328 URL: http://www.crd.york.ac.uk/PROSPERO/display_record.asp?ID=CRD42015019328

### Study inclusion and exclusion criteria

A checklist was developed to guide each reviewer. A single “no” response to any of the questions below was a cause of exclusion of the study from the systematic review:

- Does the study use SNA methods?
- Are the study subjects healthcare providers?
- Is the communication/relationship of interest between healthcare providers?
- Does the research focus on professional communication?
- Is there some metric used for performance, defined as assessing patient outcomes?

Only English search terms were used, and studies included were limited to those published in English since 1990. This date was selected in part because in the previous review, Chambers et al. 2012 had 49 of 52 included articles published after 1990. Furthermore, modern SNA studies rely on software that has primarily existed after 1990.

This review excluded studies conducting SNA of patient-to-patient communication or patient-to-provider communication. Direct-to-consumer advertising and marketing studies, such as pharmaceutical companies marketing to potential patients, were also excluded. Publication or research networks, provider patient-sharing networks, provider friendship networks, and non-empiric research were excluded. Studies whose only measures of performance were “provider perceptions” or “patient satisfaction” were excluded as not being an objectively measurable health outcome.

These exclusions were made on that basis that they were not thought to lend insight into methods used to assess professional communications among healthcare workers and their association with patient outcomes.

### Study quality assessment

Two tools were developed for critically appraising study quality—one for qualitative studies and the other one for quantitative study designs. These tools were informed by STROBE, EPOC, CASP, SIGN, ENTREQ, COREQ, RATS, QARI, and NICE Process and Methods guidelines and checklists and seminal articles on the subject [30–40]. Systematic reviews of SNAs identified through our search strategy were consulted as there is not a standard tool for assessing the quality of SNA, and some of the content of existing checklists for other study methods do not apply for network studies [5, 13, 22–29]. However the existing tools were useful starting points for assessing the quality of studies. See Additional files 5 and 6 for the tools developed to assess qualitative and quantitative studies and Table 2 for the summary of study quality. As per Cochrane and SIGN, guidance studies were assessed as being high, medium, and low quality with no summary score produced or a quality threshold for inclusion in the review [36, 41]. Mixed-methods studies had both tools applied and an overall study quality assessment provided drawing on both tools’ assessments.

Selected studies were independently critically appraised using these tools by two individuals (KS and DW). Discrepancies were discussed until reconciled.

### Data extraction strategy

A data extraction matrix was developed after reviewing data extraction tools used in relevant systematic reviews and consulting with a SNA and health expert [5, 13, 22–29]. The tool was pilot-tested and revised for greater clarity and specificity with the final version covering 35 data points. Data were extracted independently by two individuals (KS and DW), results compared and discrepancies discussed and resolved by consensus. See Additional file 7 for the tool and Tables 2, 3, 4, 5, and 6 for a subset of the data extracted.

### Data synthesis and presentation

Narrative synthesis was used to describe studies included in the review, focusing on the SNA methods and metrics used [42].

## Results

Our searches returned 5970 articles, which after double screening yielded six articles meeting our inclusion criteria [43–48]. Figure 1 documents the review process using a PRISMA flow chart.

Studies’ characteristics are described in Table 1. They were primarily recently published (all since 2010), looking at multidisciplinary healthcare providers (4 of 6) and conducted in the US (5 of 6) in tertiary care facilities or their equivalent (5 of 6).

**Table 1:** Study characteristics

Year of publication	Number	Percent
1990–2000	0	0%
2000–2010	0	0%
2010–2016	6	100%
Country		
Australia	1	17%
USA	5	83%
Type of health facility		
Hemodialysis center	1	17%
Hospital-based	3	50%
Nursing home	1	17%
Primary health care (PHC)	1	17%
Type of health professional		
Multidisciplinary teams	4	67%
Nursing staff	2	33%
Type of patients		
Emergency and outpatient department patients	1	17%
Hemodialysis patients	1	17%
Medical-surgical unit patients	1	17%
Nursing home patients	1	17%
PHC patients with alcoholism	1	17%
Renal and respiratory ward patients	1	17%
Study design		
Experimental	1	17%
Observational	5	83%
Mixed methods	2	33%
Quantitative only	4	67%
Qualitative only	0	0%

Below, we review findings based on each of our research questions.

### Primary research question

#### What SNA methods have been used to study professional communication and performance among healthcare providers?

Tables 2, 3, 4, 5, and 6 contain extractions from the six studies. Table 2 provides an overview of the studies, Table 3 focuses on their SNA methods, Table 4 lists the SNA metrics used by each study, Table 5 looks at the association between the SNA metrics and patient outcomes, and Table 6 explores the relationship between research questions and SNA methods used. Key patterns are summarized below.

**Table 2:** Summary of the six studies included in this review

Author (date)country	Study objectives	Research questions	Study design	Data collection method(s)	Critical appraisal(0/+/++)
	Type/number of healthcare worker (HCW)	Patient outcomes	Study findings	Limitations	
Effken et al. (2011) [45] USA	*Study objectives*: Identifying nursing unit communication patterns associated with patient safety and quality outcomes	*Research questions*: Can ORA’s visualizations be used to identify patient care unit network communication patterns that affect patient safety and quality outcomes?Do unit network characteristics differ by shift?What network characteristics measured by ORA metrics are related to specific safety and quality measures?	*Study design*: Observational;cross-sectional	*Data collection method(s)*: Organizational network Analysis questionnaire, previously collected survey with patient outcome data	*Critical appraisal: ++*
	*Type/number of HCW*: Nursing staff, number not stated	*Patient outcomes*: Adverse drug events, Falls, Symptom management difference, Symptom management capacity, Simple self-care management, Complex self-care management	*Study findings*: Demonstrated utility of ORA software for healthcare research and relationship of nursing unit communication patterns to patient safety and outcomes. Differences between day and night shift communication networks. Found more communication not always associated with better patient outcomes, specifically falls and adverse drug events	*Limitations*: Small and homogenous sample size. Only nursing staff. Limited to weekday shifts. Some of the patient outcomes (falls and adverse drug events) were infrequent events	
Lindberg et al. (2013) [47] USA	*Study objectives*: Evaluate how intervention affected adherence to infection prevention protocols, patient outcomes, and dialysis center social networks	*Research questions*: Does a package of interventions including membership to a collaborative emphasizing positive deviance change HCW collaboration, infection prevention, and innovation networks? Do patient outcomes change?	*Study design*: Experimental pre-post intervention Longitudinal Mixed-methods SNA retrospective	*Data collection method(s)*: Survey, focus group discussions, observation, and patient data extraction	*Critical appraisal: +*
	*Type/number of HCW*: Multidisciplinary staff at an outpatient hemodialysis facilitySNA 51 identified (46 completed each of the 2 surveys 90%)FGD 16	*Patient outcomes*: Infection rates in patients	*Study findings*: There were changes in all three networks following the implementation of the package of interventions.For collaboration: centralization and reach increased, connectivity decreased, and no change in inclusion.For bloodstream infection (BSI): prevention reach increased, the others did not change significantly.For innovation: inclusion and reach increased, reach decreased, and centralization did not change.Qualitative data supports the noted changes in network with staff looking to each other for innovations in infection prevention, working more as a cohesive team.Patient outcomes improved, with lower incidence of BSIs, although they were a relatively rare event.	*Limitations*: Results are based on one dialysis center and may not be generalizable to other centers. SNA was based on a retrospective survey and might have been subject to recall bias.The results of the time series analysis are limited as access-related bloodstream infections (AR-BSIs) are a relatively rare outcome and there were a small number of time points between interventions.Researchers were unable to stratify AR-BSIs by access type before 2009.	
Alexander et al. (2015) [43] USA	*Study objectives*: To evaluate how differences in IT sophistication in nursing homes impact communication and use of technology and associations with skin care and pressure ulcers	*Research questions*: What communication strategies do nursing home staff use to provide care to residents at risk of skin breakdown and pressure ulcers? What evidence-based pressure ulcer preventions are used by nursing home staff with diverse IT sophistication? What social networks of CNAs enhance or interrupt workflow and have positive or negative effects on nursing work?	*Study design*: Observational mixed-method case studies	*Data collection method(s)*: For SNA, observation of communication among HCWs was documented using a structured field note guide.	*Critical appraisal: +*
	*Type/number of HCW*: Nursing staff, FGD, 21;SNA, nurses at 2 nursing homes, 1386 observations (unit of analysis was not based on number of HCWs)	*Patient outcomes*: Incidence of pressure ulcers	*Study findings*: High IT sophistication lead to more diverse locations for HCW interactions. Low IT sophistication required more face to face interaction in more centralized locations within the nursing home. Patient outcomes captured were more or less equivalent between the two facilities.	*Limitations*: The study focused on observations only during the day shift. Individual RNs/LPNs and CNAs were not uniquely identified during observations, and the analysis lumped them together as RNs/LPNs and CNAs, respectively. Two nursing homes with a specific degree of IT sophistication were compared, rather than following any change due to the introduction of IT sophistication. Confounding variables offer an opportunity for increasing bias in the results. Generalizability may not be appropriate as this study was an in-depth analysis of two nursing homes in one state––Missouri.	
Creswick and Westbrook (2015) [44] Australia	*Study objectives*: Determine if there are network property differences in prescription advice-seeking associated with prescription errors	*Research questions:* 1. Identify and measure from whom hospital clinical staff seek medication advice on a weekly basis 2. Quantify the use of other sources of medication information, assess the difference in medication advice-seeking patterns across professional groups 3.Examine network characteristics in relation to prescribing error rates	*Study design*: Observational Cross-sectional	*Data collection method(s)*: Questionnaire for SNA and clinical audit	*Critical appraisal: ++*
	*Type/number of HCW*: Multidisciplinary: physicians, nurses, and allied health professionals101 participants	*Patient outcomes*: Prescription error rates	*Study findings*: Limited interprofessional advice-seeking overall (particularly between physicians and nurses). Hubs of advice provisions include pharmacists, junior physicians, and senior nurses. Senior physicians are not involved in these advice exchange networks. The ward with the stronger (denser) advice-seeking network had lower rates of procedural and clinical prescribing errors.	*Limitations*: Limited in scope (only two wards).No psychometric assessments. Networks only examined at one point in time.	
Mundt et al. (2015) [48] USA	*Study objectives*: To understand what team communication structures contribute to alcohol-related utilization of care and medical costs.	*Research questions*: What primary care team communication networks are associated with alcohol-related utilization of care and medical costs for primary care patients?	*Study design*: Observational Retrospective	*Data collection method(s)*: Questionnaire administered in person, electronic health record extractions	*Critical appraisal: ++*
	*Type/number of HCW*: Multidisciplinary: physicians, physician assistants, nurse practitioners, registered nurses, medical assistants, licensed practical nurses, laboratory technicians, radiology technicians, clinic managers, medical receptionists, and other patient care staff.One hundred sixty HCWs were invited, 155 took part; 31 care teams	*Patient outcomes*: Alcohol-related emergency department visits,Hospital days and associated costs	*Study findings*: Teams’ variations in communication patterns (face to face and through electronic health record) are associated with statistically significant differences in alcohol-related patient utilization and medical costs in their patient panels. Excessive alcohol-using patients may fair better if they are cared for by teams with RNs who interact with more team members including LPNs/MAs and by teams whose frequent daily face-to-face communication to the primary care practitioner has been streamlined to a smaller number of team members.	*Limitations*: Only six practices in limited geography included. No information on content of communication. No information on frequency and quality of alcohol services delivered. Unclear rationale for type of communication method. Increased risk of type I error. Study may underestimate full impact given underreporting of alcohol-related diagnoses in electronic health record.	
Hossain and Guan (2012) [46] United States	*Study objectives*: To understand coordination in an emergency department through measures ofperformance and quality	*Research questions*: Test the following hypotheses: Performance of coordination in the emergency department is influenced by the social network. Performance of coordination in the emergency department is influenced by the centrality of the network. Performance of coordination in the emergency department is influenced by the density of the network. Performance of coordination in the emergency department is influences by the degree of connections in the network.	*Study design*: Observational Cross-sectional	*Data collection method(s)*: National Hospital Ambulatory Medical Care Survey (NHAMCS), patient record surveys selected from emergency departments	*Critical appraisal: ++*
	*Type/number of HCW*: Multidisciplinary: emergency department hospital staff. Staff included in patient reports from 359 emergency departments	*Patient outcomes*: Length of visit, wait time to see physician, revisits within 72 h, deaths within emergency department, and left before seeing physician	*Study findings*: Coordination and the social network are heavily related within the emergency department. Specifically, as emergency department network density increases, number of patients waiting over triage time decreases but does not influence average wait times. As degree of connection increases, the wait time for patients increases. No evidence of connection between quality of service and death and the social networks. Quality of coordination in emergency department is influenced by centrality of the network. As communication in emergency department increases, the number of patients revisiting decreases.	*Limitations*: NHAMCS dataset is incomplete, contains less than 40 surveys of each emergency department, which is less than the assumed volume of patients in a 3-month period.

**Table 3:** Summary of studies’ social network analysis methods

Authors	Effken et al.	Hossain and Guan	Lindberg et al.	Alexander et al.	Creswick and Westbrook	Mundt et al.
Data collection method	Network survey, previously collected survey with patient outcomes	Extraction from National Hospital Ambulatory Medical Care Survey (NHAMCS), patient record surveys selected from emergency departments	Survey, focus group discussions, observation, and patient data extraction	Observation, previously collected survey	Network survey and clinical audit	Network survey and electronic health record extractions
Boundary specification method/sampling (if applicable)	All nursing staff who worked in one of seven patient care units in three magnet hospitals	Emergency departments of 359 hospitals responded to the ambulatory survey section of NHAMCS survey conducted by the CDC.	All staff at 21 hemodialysis facilities that form part of the CDC Hemodialysis BSI Prevention Collaborative	Comparative case study of two units within two nursing homes, one with the highest IT sophistication and one with the lowest IT sophistication based on a statewide census in 2007. Nodes were both HCWs and the locations and content of their interactions.	All HCWs in two wards	Eight clinics in Southern Wisconsin were invited to participatein the study, and six agreed. Sites were chosen based on consultation with leadership from the healthcare system.
Network category studied 1. Whole, ego, or hybrid network 2. Directed or undirected 3. Valued or dichotomous	1. Whole network 2. Directed 3. Valued	1. Whole network 2. Directed 3. Dichotomous	1. Whole network 2. Directed 3. Valued	1. Whole network 2. Directed 3. Valued	1. Whole network 2. Directed 3. Valued	1. Whole network 2. Directed 3. Valued
Response rate	Not stated	N/A as SNA data extracted from surveys on patients	90%	N/A as SNA from observation	90%	97%
Network metrics used	Clustering coefficient, component count strong, component count weak, density, diffusion, fragmentation, hierarchy, isolates, in-degree centrality, out-degree centrality, eigenvector centrality, simmelian ties, betweenness centrality, number of triads, and number of cliques	SNA metrics: degree, density, and centrality	Connectivity, inclusion, reach, and centralization	None	Density Reciprocity In-degree centrality	In-degree centrality, tie strength
Analyses conducted	Correlations (Spearman Rho) calculated between SNA metrics and patient outcomes	Multiple linear regression, *p* values and *r* values reported	Quantitative: Pearson *X* ^2^ and Fisher’s exact test, *t* test. Reported *p* valuesQualitative analysis: reflexive observation and contextual analysis	Quantitative: calculated highest and lowest ITS NH from survey data in an earlier study Qualitative: axial coding, themes developed using human factors theory	Chi-squared with *p* values	Linear modeling (GLMM) and sensitivity analyses
Software	ORA, Excel	UCINET, SPSS, Excel	Not stated	ORA, Nvivo, Excel	UCINET and NetDraw	UCINET, HLM 7.0
Network map (yes/no)	Yes	Yes	Not stated	Yes	Yes	Yes
Further research	Replicate study, expand to larger, more diverse group of patient care units. Consider shifting to more patient-centric focus, including full team of care providers	Further research needed to verify the relationship suggested by this study between coordination and social network analysis. Survey of emergency departments within Australia for a period of 1 year, to allow accurate measurements to be taken and utilized for the study and for verifying the relationship between social networks and coordination in an emergency department.	None stated	To demonstrate how organization analytics about communication can be used to benchmark evidence-based practices	Further research on link between medication advice-seeking networks and errors, as this study suggests. Also, whether the increased use of electronic medication management systems means that information needs are met through channels other than communication between physicians, nurses and pharmacists, or that information sharing regarding medication issues is reduced and may impact medication safety. Evaluate interventions to engage senior physicians in advice exchange networks. Further health applications of SNA surveys needed to improve validity and reliability of tools.	Longitudinal and experimental studies needed to explore the causal pathways between team communication variables and alcohol-related patient care
Network intervention (yes/no)	No	No	Yes (although intervention not based on baseline network analysis. Rather, it was developed with the intention of changing HCW networks)	No	No	No

**Table 4:** Summary of studies’ social network analysis metrics

Social Network Analysis Metric	Effken et al.	Lindberg et al.	Alexander et al.	Creswick and Westbrook	Mundt et al.	Hossain and Guan	Total
Centralization		X					1
Centrality (in-degree)	X				X	X	3
Centrality (out-degree)	X						1
Centrality (eigenvector)	X						1
Centrality (betweenness)	X						1
Clustering coefficient	X						1
Component count strong	X						1
Component count weak	X						1
Connectivity		X					1
Degree						X	1
Density	X			X		X	3
Diffusion	X						1
Fragmentation	X						1
Hierarchy	X						1
Inclusion		X					1
Isolates	X						1
Number of triads	X						1
Number of cliques	X						1
Reach		X					1
Reciprocity				X			1
Simmelian ties	X						1
Tie strength					X		1
Total	15	4	0	2	2	3	26

**Table 5:** Analysis of studies’ SNA metrics and patient outcome findings

**Metric**	**Study**	**Patient outcomes**	**Association with metric**	**Overall association**
Centrality	Effken et al.	Adverse drug events (ADEs)	“Betweenness centrality” positively correlated (rho = .73) with ADEs	Generally, as centrality measures increase, patient outcomes improve; however, there were many patient outcomes for which there was no significant association with a centrality measure. Effken exception. Higher betweenness centrality, with potentially more gatekeepers resulted in more ADEs. With symptom management difference, the seemingly inconsistent association with centrality could actually point to the importance of small group communication with this outcome measure and that those with more out-degree ties are novices seeking advice.
		Falls	Not significant	
		Symptom management difference	“Centrality out-degree” negatively correlated (rho = −.79) although eigenvector centrality positively correlated (rho = .69)	
		Symptom management capacity	Not significant	
		Simple self-care management	Not significant	
		Complex self-care management	Not significant	
	Lindberg et al.	Access-related bloodstream infections	Not significant	
	Mundt et al.	Alcohol-related emergency department visits	Statistically significant (sig.) GLMM model with only weak “in-degree ties” had positive association(RR 1.23, *p* < 0.01), models with any strong ties had inverse association (RR range 0.8–0.9, *p* < 0.05)	
		Alcohol-related hospitalizations	Sig. GLMM models with groups of HCWs with any weak “in-degree ties” had positive association (RR 1.1, *p* < 0.05, RR 1.25, *p* < 0.01), model with groups of HCWs with only strong ties had inverse association (RR .95, *p* < 0.05)	
		Alcohol-related costs per 1000 team patients over 12 months	In an average team size of 19, the addition of a HCW with strong “in-degree ties” reduced cost by $1030 (*p* < 0.05),weak ties increased cost by $2922 (*p* < 0.01)	
	Hossain and Guan	Wait time to see physician	Not significant	
		Revisits within 72 h	Not significant	
		Deaths within emergency department	Not significant	
		Left before seeing physician	“Network centralization” inversely associated (beta = − 0.221, sig. < 0.001)	
**Metric**	**Study**	**Patient Outcomes**	**Association with metric**	**Overall association**
Density	Effken et al.	Adverse drug events	Not significant	Density positively associated with improved patient outcomes. However, there were patient outcomes for which there was no significant relationship with density.
		Falls	Not significant	
		Symptom management difference	Positively associated (rho = 0.70, *p* < 0.10)	
		Symptom management capacity	positively associated (rho = 0.75, *p* < 0.10)	
		Simple self-care management	Not significant	
		Complex self-care management	Not significant	
	Creswick and Westbrook	Prescription error rates (procedural and clinical)	Inversely associated (ward A error rates 5.46 and 1.81 with density 12% vs ward B error rates 1.53 and 0.63 with density 7%)	
	Hossain and Guan	Wait time to see physician	Inversely associated (beta = − 0.107) for waiting “overestimated triage time” but not significant for “waiting above average”	
		Revisits within 72 h	Inversely associated (beta = − 0.159, sig. = 0.003)	
		Deaths within emergency department	Not significant	
		Left before seeing physician	Inversely associated (beta = − 0.273, sig. < 0.001)

**Table 6:** Analysis of studies’ research questions and study methods used

Study	Objectives/research questions	Research question categories	Methods
Effken et al.	Identify nursing unit communication patterns associated with patient safety and quality outcomes1. Can ORA’s visualizations be used to identify patient care unit network communication patterns that affect patient safety and quality outcomes?2. Do unit network characteristics differ by shift?3. What network characteristics measured by ORA metrics are related to specific safety and quality measures?	1. Descriptive 2. Descriptive 3. Relational	*Design*: observational, cross-sectional *Data collection*: Organizational Network Analysis questionnaire, patient outcome survey *Analyses*: correlations(Spearman Rho) calculated between SNA metrics and patient outcomes
Lindberg et al.	Determine if intervention changed adherence to infection prevention protocols, patient outcomes and dialysis center social networks 1. Does a package of interventions including membership to a collaborative emphasizing positive deviance change HCW collaboration, infection prevention and innovation networks? 2. Do patient outcomes change?	1. Causal 2. Causal	*Design*: experimental, longitudinal, mixed methods, pre-post intervention *Data collection*: survey, FGD, observation, patient data extraction *Analyses*: quantitative: Pearson *X*2 and Fisher’s exact test, *t* test Reported *p* valuesQualitative analysis: reflexive observation and contextual analysis
Alexander et al.	Evaluate how differences in IT sophistication in nursing homes impact communication and use of technology related to skin care and pressure ulcers. 1. What communication strategies do nursing home staff use to provide care to residents at risk of skin breakdown and pressure ulcers? 2. What evidence-based pressure ulcer preventions are used by nursing home staff with diverse IT sophistication? 3. What social networks of CNAs enhance or interrupt workflow and have positive or negative effects on nursing work?	1. Descriptive 2. Descriptive 3. Relational	*Design*: observational mixed methods, case studies *Data collection*: observation, previously collected survey *Analysis*: quantitative: calculated highest and lowest ITS NH and patient outcomes from survey data in an earlier study Qualitative: axial coding, themes developed using human factors theory
Creswick and Westbrook	Determine if there are network property differences in prescription advice-seeking associated with prescription errors 1. Identify and measure from whom hospital clinical staff seek medication advice on a weekly basis 2. Quantify the use of other sources of medication information, assess the difference in medication advice-seeking patterns across professional groups 3. Examine network characteristics in relation to prescribing error rates	1. Descriptive 2. Descriptive 3. Relational	*Design*: observational, cross-sectional *Data collection*: network survey and clinical audit *Analyses*: chi-squared with *p* values
Mundt et al.	To understand what team communication structures contribute to alcohol-related utilization of care and medical costs 1. What primary care team communication networks are associated with alcohol-related utilization of care and medical costs for primary care patients?	1. Relational	*Design*: observational, cross-sectional, retrospective *Data collection*: network survey, electronic health record extractions *Analyses*: linear modeling (GLMM) and sensitivity analyses
Hossain and Guan	To understand coordination in an emergency department through measures of performance and quality 1. Is performance of coordination in the ED influenced by the social network? 2. Is performance of coordination in the ED influenced by the centrality of the network? 3. Is performance of coordination in the ED influenced by the density of the network? 4. Is performance of coordination in the ED influenced by the degree of connections in the network?	1. Causal 2. Causal 3. Causal 4. Causal	*Design*: observational, cross-sectional *Data collection*: survey extraction *Analyses*: multiple linear regression, *p* values and *r* values reported

All studies included in this review were exploratory in nature. All but one study [47] employed a cross-sectional study design looking at whole networks. Four studies could only look at one or two whole networks, listing this as a limitation to their study’s generalizability [43–45, 47]. Data collection tools were typically network surveys, designed specifically for that study. However, one study coded observations [43] and another study extracted data on healthcare worker communications from surveys of patients that attended emergency departments [46]. All but one study [47] visualized their networks, which is not surprising as one of the unique aspects of SNA methods and software is the ability to visualize networks. Software preferences leaned towards UCINET and ORA [49, 50] with Microsoft Excel and SPSS mentioned as supplementary tools. A wide range of network metrics were calculated, although density and centrality were the most commonly calculated. See Table 4 below for an overview of which studies calculated specific network metrics. There was a range in how these data were analyzed with some integrating them into models and others using tests of significance.

### Secondary research questions

#### What is the quantity of SNA studies? What was the evolution over time?

Six studies were identified, with all studies published in the past 5 years and none before 2011. One study was published annually from 2011 to 2013, and then, three were published in 2015. The evolution over time suggests there is an increasing interest in this type of study; however, with only six studies, it may be a premature assessment.

#### To what extent has this research taken place in low- and middle-income countries?

Not a single study that met our search criteria was conducted in a low- or middle-income country. All studies took place in either the USA [43, 45–48] or Australia [44].

#### What is the quality of these studies?

The quality of the studies meeting our selection criteria was assessed using the tools found in Additional files 5 and 6 and summarized in Table 2. None of the studies were found to be of low quality, two were found to be of acceptable quality and four of high quality applying the SIGN guidelines for assigning these categories.

#### What methods were used for which types of research questions?

There were only two studies using mixed methods, the other four only used quantitative methods.

Interestingly, Alexander et al. [43] only visualized the communication networks and did not calculate SNA metrics. Their authors cited this as a limitation of their design, which did not involve coding the observations in a way that would allow for healthcare workers to be individual nodes. Yet another study, Lindberg et al. [47], did not include a visualization of the communication network.

Modeling and tests of significance were used when studies intended to measure the association of network properties with other factors. Table 2 lists the study objectives, research questions, study design, and data collection methods whereas Table 3 goes into detail regarding each study’s SNA methods. Table 4 summarizes the SNA metrics used by each study, and Table 6 looks at the link between research questions and study design.

#### What are the main limitations of the SNA methods?

Table 2 lists the limitations, identified by our reviewers (although some were mentioned by the authors as well) of each of the six studies. While some of these limitations do not relate to the SNA methods, they provide insight into some of the challenges faced by studies using such methods. A general challenge generated by SNA methods is the need to clearly define the study boundary, which can limit sample size and therefore affect the broader generalizability. This came up in several studies noting areas for further research including broadening to other settings and repeating the study elsewhere given the limited sample size. The sample size limitation related less to the number of nodes, but more to the number and type of whole networks included.

The lack of longitudinal and experimental designs speaks to a broader challenge in the field as these are new areas for application of SNA methods, and the analytical tools and software are still in development. This limited the ability of SNA studies to address causal pathways.

Similarly, the limited qualitative methods being integrated into the studies constrain the contextual understanding of the network properties quantified and visualized through applying the quantitative SNA methods.

One study, Alexander et al. [43] reported that their coding method limited the type of analyses that can be conducted, and therefore, they did not analyze their SNA data beyond visualizing patterns.

#### To what extent has this research focused on community-based health providers?

None of the studies took place in a community-based setting. Only one study took place in a primary healthcare context [48]. The other five studies took place in tertiary level facilities including hospital units and specialist care facilities like hemodialysis centers or nursing homes [43–47].

#### What are the key findings of these SNA studies?

While this is a methodologically focused review, therefore inherently less focused on any associations observed between network properties and the health outcomes measured, the next question naturally arises, what did these studies find? Their overall study findings are discussed in Table 2, but to better understand any relationship between specific network metrics and patient health outcomes, we looked at the metrics captured in more than one of the studies and their reported association with patient outcomes in Table 5. There were only two metrics, density, and in-degree centrality reported in more than one study. For this analysis, all centrality metrics were collapsed into one category, although the actual centrality metrics used in the study are specified in Table 4.

Patient outcomes generally improved when healthcare worker communication was denser and more centralized as measured by various centrality metrics. However, for both metric studies reported no significant association with some patient outcomes, as such more studies are needed to clarify patterns.

The Effken study had one exception to the relationship proposed between centrality and patient outcomes. Adverse drug events increased with betweenness centrality, possibly due to the presence of gatekeepers the authors hypothesized. Another patient outcome, symptom management on the surface appears to have conflicting associations with centrality metrics; however, the authors suggest that taken together the correlation of this patient outcome metric with eigenvector centrality and patient symptom management capacity with simmelian ties (strong ties within cliques) which could point to the importance of small group communication [45]. This broader pattern of performance being linked to more centralized networks is generally supported by the SNA literature, although the debate continues [51]. Furthermore, patient outcomes may not necessarily be expected to be associated with healthcare provider centrality as they could be central for reasons other than the quality of care or professional advice they provide. Network density can provide more pathways for communication, however, in its extreme, can reinforce insularity and limit external sources of information [51]. As such a network diagnostic tool proposed ideal network density to be .15–.50 [12].

There are definite limitations to this specific analysis. Through this process, it became clear that not every SNA metric calculated and its association with all outcomes captured in a study are published. Another complication is that not all of the metrics and results were truly comparable given the different data sources and analytical approaches. For example, one study used GLMM models collapsing all data collected across teams that had different professionals with either strong or weak ties for two types of communication networks (electronic and face to face) rather than looking at the in-degree centrality of a network and its association with the patient outcome of interest.

## Discussion

The discussion will focus on the two main research questions of the review, the primary research question “What SNA methods have been used to study professional communication and performance among healthcare providers?” and “What methods were used for which types of research questions?”

### What SNA methods have been used to study professional communication and performance among healthcare providers?

The majority (5 of 6) of the studies that met our selection criteria used a cross-sectional, observational study design. This posed challenges in addressing the research questions looking at the association between provider communication networks and patient outcomes, as the patient outcome data timeframe and the networks being captured, were not always temporally aligned.

As other systematic reviews suggested, there remain few network intervention studies, a frontier opportunity for researchers [5, 26, 27]. See Additional file 8 for an overview of the other SNA and health systematic reviews identified through our search strategy and their recommendations for further research. The lone experimental study included in this review, Lindberg et al.*,* did not use network data to design the intervention, so it does not qualify as a “network intervention” [47]. Network interventions and experimental, longitudinal study designs will allow for SNA methods to address causal pathways, a current limitation on how the methods are being applied [29].

One of the challenges facing researchers wanting to use SNA methods is the lack of validated SNA survey tools for use in the health sector, as highlighted by Creswick and Westbrook and Perkins et al. [29, 44]. While this only is relevant for those interested in using sociometric survey methods, as more studies use SNA methods, we can anticipate that a set of tools or best practices for applying a range of SNA methods will emerge. The Perkins systematic review aimed to address one aspect of that gap by gathering all the name generating tools they found across the studies they reviewed [29]. However, this is only one step in the process of having more systematically validated tools and best practices available.

The most obvious pattern in study methods was that studies looking to establish associations used more advanced statistical methods to test their hypotheses whereas the studies that were looking to answer questions about processes used more qualitative methods. However, this observation is largely less about SNA methods and more about the relative strengths of qualitative vs quantitative research methods. The diversity of SNA analytical methods could also speak to the expertise of the individual researchers and which methods they were more comfortable using rather than necessarily a clear advantage posed by using one method over another to answer a given research question. That said there are SNA methods such as exponential random graph models which are appropriate to answer specific SNA questions that other SNA methods would not be able to address. These methods were not used in the studies meeting our search criteria.

It is important to note that while this review did not identify any studies conducted in LMIC, this does not mean SNA methods have never been used to study health in these contexts. A systematic review looked specifically at SNA applications in LMIC and found 17 articles from 10 health-related network studies; however, their focus was broad and none of the studies met our criteria of focusing on healthcare provider communication and patient outcomes [29]. Instead, these studies set in 9 countries looked primarily at patients or their household as the ego and used name generators to establish networks related to contraception use and family planning, mercury consumption (2 studies), HIV transmission (5 studies), and diarrheal disease transmission (3 studies) [29].

One of the issues with the way SNA methods have been applied in the health sector is the often artificial boundaries imposed by limiting studies to specific cadres, which did not reflect the actual care environments. Notably four of six studies looked at multidisciplinary teams and one of the studies limited to one cadre, Effken, et al. [45], suggested that future studies look at other providers in the care setting.

There was a surprising variability across the studies with respect to the network metrics calculated and used. Two—centrality (in-degree) and density––were included in 4 and 3 of 6 studies. The range of network metrics calculated per study included in our review ranged from 0 to 15 with most calculating 3 or 4. See Table 4 for a breakdown of which studies calculated which metrics.

### What methods were used for which types of research questions?

With only six studies meeting our criteria, there are limits to identifying clear patterns in methods used to address types of research questions and study objectives. Table 6 focuses on the link between types of research questions and study methods. Research questions were classified as either descriptive, relational, or causal in nature [52]. Half of the studies included more than one type of research question. Those studies that included causal or relational research questions typically involved more robust quantitative analyses. Mixed methods were used in two studies: one only had causal research questions while the other had descriptive and relational research questions. Most study designs were observational and cross-sectional and had descriptive and relational research questions. As more studies are conducted over the coming years, these patterns will likely evolve and become more consistent.

While the focus of our review has been on these two research questions as applied to the six articles that met our search criteria, there are a range of SNA methods and metrics beyond what is discussed here which could have applicability in answering research questions related to healthcare professional advice networks and performance including, but not limited to block modeling, core-periphery, presence of structural holes and bridges, cohesion, proximity, and prestige/prominence analyses.

### Limitations of the review

This review looked at a very specific question and found that few, albeit in recent years a growing number of researchers, have designed studies meeting these criteria. Our definition of performance as being assessed by patient outcomes rather than through proxy interim measures, such as use of evidence-based tools and practices, restricted the studies that met our search criteria. This may have been particularly limiting for studies of community-based healthcare, which often takes the form of counseling whereby certain outcomes like patient satisfaction are more likely to be appropriate study outcomes than patient outcomes. Our definition of professional communication networks excluded studies of provider friendship networks or other types of ties between healthcare workers unless they explicitly captured professional communication. In theory, those networks may have embedded professional advice exchange not captured, analyzed, or presented in the paper. Another limitation is that we only looked at English language publications. However, looking at other systematic reviews of SNA studies, that is a common limitation [13, 22, 25, 27–29]. For those that included studies in other languages, like Benton et al. which included Spanish and Portuguese language studies, they found 2 of 43 included studies were non-English and excluded a further 2 for language reasons [23]. Chambers et al. and Flodgren et al. [5, 26] did not impose any language restrictions in their searches but did not identify studies published outside of English language journals, so this is unlikely to be a major source of bias. We limited our searches to those studies published from 1990, although given the emphasis on software packages in current SNA studies, it was believed that few studies would have been using relevant methods before 1990 as those software packages did not exist for use on widely accessible platforms.

Another limitation speaks to broader limitations of systematic reviews. The language used for social network analysis is vague and inconsistent, and search strategies were challenging to devise that returned a manageable number of articles to screen yet were broad enough to capture all the ways in which researchers may have described an SNA study.

## Conclusion

Five years after the Chambers et al.’s [5] review, searched for articles, social network analysis methods continue to be underutilized in the health sector, particularly when looking at healthcare provider communication and performance. There are few studies that do more than describe professional communication networks among healthcare providers, for those that do, only a small subset, six measure performance using patient outcomes. This may be a broader reflection of the challenge in accurately capturing patient outcome data as many studies were excluded for using proxy measures such as patient satisfaction or use of an evidence-based practice. While a diverse set of methods were used across the six studies, as more studies are conducted clearer patterns in methods may emerge. The quality of these studies was either acceptable or high; however, the level of sophistication of these studies was relatively low with an emphasis on cross-sectional study designs. This is not an unsurprising finding as the network methods themselves and software tools capable of dynamic and longitudinal network analyses are still developing. As longitudinal SNA analysis methods mature, other study designs and network interventions should become more common. All articles meeting the review criteria were published in the past 5 years, suggesting that this is a developing area of research.

One pattern that this review highlights is a trend towards looking at multidisciplinary provider networks rather than focusing on one cadre. Other SNA methodological consistencies among these six studies included a preference for calculating specific network metrics: density and centrality. The limited number of articles meeting our search criteria, the glaring lack of any studies in LMIC, non-Western contexts, and in non-tertiary settings or community-based settings present clear research opportunities. Once there are more studies published addressing healthcare provider communication and performance, it may be useful to revisit this analysis and draw conclusions on the SNA methods best placed to answer specific research questions within this space.

## Additional files

Additional file 1: Search strategy concepts and associated terms. (XLSX 12 kb)

Additional file 2: SNA software search strategy for each database. (XLSX 10 kb)

Additional file 3: K* search strategy development. (DOCX 107 kb)

Additional file 4: MEDLINE search strategy. (DOCX 18 kb)

Additional file 5: Critical appraisal tool for qualitative studies. (XLSX 13 kb)

Additional file 6: Critical appraisal tool for quantitative studies (XLSX 15 kb)

Additional file 7: Data extraction tool. (XLSX 11 kb)

Additional file 8: Existing SNA systematic reviews. (XLSX 14 kb)

## Abbreviations

ADE: Adverse drug event
AR-BSI: Access-related bloodstream infection
BSI: Bloodstream infection
CASP: Critical Appraisal Skills Programme
CDC: Centers for Disease Control and Prevention
CNA: Certified Nursing Assistant
COREQ: Consolidated criteria for reporting qualitative research
DOI: Digital Objective Identifier
ED: Emergency department
ENTREQ: Enhancing transparency in reporting the synthesis of qualitative research
EPOC: Effective Practice and Organisation of Care
FGD: Focus group discussion
GLMM: Generalized linear mixed model
HCW: Healthcare worker
IT: Information technology
ITS: Information technology sophistication
LMIC: Low- and middle-income countries
LPN: Licensed practical nurse
MA: Medical assistant
NH: Nursing home
NHAMCS: National Hospital Ambulatory Medical Care Survey
ORA: Organization Risk Analyzer
PRISMA: Preferred Reporting Items for Systematic reviews and Meta-Analyses
PROSPERO: An international database of prospectively registered systematic reviews in health and social care, welfare, public health, education, crime, justice, and international development
QARI: Qualitative assessment and review instrument
RATS: Relevance of study question, appropriateness of qualitative method, transparency of procedures, and soundness of interpretive approach
RN: Registered nurse
SIGN: Scottish Intercollegiate Guidelines Network
SNA: Social network analysis
STROBE: Strengthening the Reporting of Observational studies in Epidemiology
