# Supplementary material for: Use of social network analysis methods to study professional advice and performance among healthcare providers: a systematic review
Source: Syst Rev. 2017 Oct 23;6:208. doi: 10.1186/s13643-017-0597-1 (PMC5651641; doi:10.1186/s13643-017-0597-1)
Supplement: Supplementary file 3 — K* search strategy development. (DOCX 107 kb) [file 13643_2017_597_MOESM3_ESM.docx]

**Additional File 3: K* Search Strategy Development**

Terms appearing in two or more sources were included in the search strategy. Those highlighted in blue technically only appeared in one source, however upon further investigation another iteration of the term (with an adjacency factor) appears in another source, therefore they are included. Those highlighted in orange were included in the Diffusion of Innovation search strategy. Depending on the database additional terms were included to ensure more litmus test articles were included in the search. In some databases some of the following terms yielded thousands and in some cases, millions of off topic articles, in those cases the terms were removed from the strategy. For example, “adoption” in most databases referred to adoption of children, rather than adoption of innovations. Therefore the term “adoption” was removed in those situations. Other terms that were removed in certain cases included “change”, “use”, “utilization”, “information” and “policy”. All changes were documented in a search log.

| **K* Term** | **KT Wiki** | **LaRocca** | **McKibbon** | **Mitton** | **Scott** | **Van Eerd** | **Count** |
| --- | --- | --- | --- | --- | --- | --- | --- |
| **Action Research** | **X** |  |  |  |  | **X** | **2** |
| **Adoption** | **X** | **X** | **X** |  |  |  | **3** |
| **Applied Dissemination** | **X** |  |  |  |  | **X** | **2** |
| **Best practi?e** | **X** | **X** | **X** |  |  |  | **3** |
| **Change** | **X** | **X** | **X** |  |  |  | **3** |
| [**Communit$ of practice**](mailto:Communit@%20of%20practice) | **X** |  | **X** |  |  |  | **2** |
| **Complex intervention** |  | **X** | **X** |  |  |  | **2** |
| **Continuing Education** | **X** | **X** | **X** |  |  |  | **3** |
| **Diffusion** | **X** |  | **X** |  |  |  | **2** |
| **Diffusion of Innovation** | **X** | **X** | **X** |  | **X** | **X** | **5** |
| **Dissemination** | **X** | **X** | **X** |  |  |  | **3** |
| **Effective dissemination** | **X** |  |  |  |  | **X** | **2** |
| **Evaluation** | **X** | **X** | **X** |  |  |  | **3** |
| **evidence adj2 diffusi$** |  |  |  |  | **X** | **X** | **2** |
| **evidence adj2 implement$** |  |  |  |  | **X** | **X** | **2** |
| **evidence adj2 tranlsat$** |  |  |  |  | **X** | **X** | **2** |
| **evidence adj2 utili#$** |  |  |  |  | **X** | **X** | **2** |
| **Evidence-based medicine** | **X** |  |  |  |  | **X** | **2** |
| **Evidence-based practice** | **X** |  |  |  |  | **X** | **2** |
| **K* Term** | **KT Wiki** | **LaRocca** | **McKibbon** | **Mitton** | **Scott** | **Van Eerd** | **Count** |
| **Implementation** | **X** | **X** | **X** |  |  |  | **3** |
| **Implementation adj3 research** |  | **X** | **X** |  |  |  | **2** |
| **Implementation research** | **X** | **X** |  |  |  | **X** | **3** |
| **Implementation science** | **X** |  |  |  |  | **X** | **2** |
| **information** | **X** | **X** | **X** |  |  |  | **3** |
| **innovation** | **X** | **X** | **X** |  |  |  | **3** |
| **Institutionali#ation** | **X** | **X** | **X** |  |  |  | **3** |
| **Integrated knowledge translation** | **X** |  |  |  |  | **X** | **2** |
| **knowledge adj2 diffusion** |  |  |  |  | **X** | **X** | **2** |
| **knowledge adj2 dissemination** |  |  |  |  | **X** | **X** | **2** |
| **knowledge adj2 transfer** |  |  |  |  | **X** | **X** | **2** |
| **knowledge adj2 uptak$** |  |  |  |  | **X** | **X** | **2** |
| **knowledge adj2 utili#$** |  |  |  |  | **X** | **X** | **2** |
| **Knowledge broker$** |  |  |  | **X** |  | **X** | **2** |
| **Knowledge exchange** | **X** |  |  | **X** |  |  | **2** |
| **Knowledge generation** |  |  |  | **X** |  | **X** | **2** |
| **Knowledge integration** | **X** |  |  |  |  | **X** | **2** |
| **Knowledge management** | **X** |  | **X** |  |  |  | **2** |
| **Knowledge mobli#ation** | **X** |  |  | **X** |  | **X** | **3** |
| **Knowledge to action** | **X** |  |  |  |  | **X** | **2** |
| **Knowledge transfer** | **X** |  |  | **X** |  |  | **2** |
| **Knowledge translation** | **X** |  |  | **X** |  |  | **2** |
| **Knowledge uptake** | **X** |  |  | **X** |  |  | **2** |
| **Linkage and exchange** | **X** | **X** | **X** |  |  | **X** | **4** |
| **Organizational innovation** | **X** | **X** | **X** |  | **X** |  | **4** |
| **Participatory Action Research** | **X** |  | **X** |  |  |  | **2** |
| **policies** |  | **X** | **X** |  |  |  | **2** |
| **Policy** | **X** | **X** | **X** |  |  |  | **3** |
| **K* Term** | **KT Wiki** | **LaRocca** | **McKibbon** | **Mitton** | **Scott** | **Van Eerd** | **Count** |
| **Policy research** | **X** |  |  |  |  | **X** | **2** |
| **Quality Improvement** | **X** | **X** | **X** |  |  |  | **3** |
| **research adj2 implement$** |  |  |  |  | **X** | **X** | **2** |
| **research adj2 transl$** |  |  |  |  | **X** | **X** | **2** |
| **research adj2 utilizat$** |  |  |  |  | **X** | **X** | **2** |
| **Service innovation** | **X** | **X** | **X** |  |  |  | **3** |
| **Total Quality Managment** | **X** |  | **X** |  |  |  | **2** |
| **Translational research** | **X** | **X** | **X** |  |  |  | **3** |
| **Use** | **X** |  | **X** |  |  |  | **2** |
| **Utili#ation** | **X** | **X** | **X** |  |  |  | **3** |
| **Applied Health Research** | **X** |  |  |  |  |  | **1** |
| **Audit** | **X** |  |  |  |  |  | **1** |
| **Behavi?or$ adj2 chang$** |  |  |  |  |  | **X** | **1** |
| **Behavio?r utili#ation** | **X** |  |  |  |  |  | **1** |
| **Business case** |  |  |  |  |  | **X** | **1** |
| **Capacity building** | **X** |  |  |  |  |  | **1** |
| **Change implementation** | **X** |  |  |  |  |  | **1** |
| **Clinical and translational science** | **X** |  |  |  |  |  | **1** |
| **Cognitive Application** | **X** |  |  |  |  |  | **1** |
| **Collaborative Development** | **X** |  |  |  |  |  | **1** |
| **Communication** | **X** |  |  |  |  |  | **1** |
| **Communicative utilization** | **X** |  |  |  |  |  | **1** |
| **Communit$ based research** |  |  |  |  |  | **X** | **1** |
| **Comparative effectiveness research** | **X** |  |  |  |  |  | **1** |
| **Complexity Science** | **X** |  |  |  |  |  | **1** |
| **Conceptual utili#ation** | **X** |  |  |  |  |  | **1** |
| **Confirmatory utili#ation** | **X** |  |  |  |  |  | **1** |
| **K* Term** | **KT Wiki** | **LaRocca** | **McKibbon** | **Mitton** | **Scott** | **Van Eerd** | **Count** |
| **Continuing Medical Education** | **X** |  |  |  |  |  | **1** |
| **Continuing Nursing Education** | **X** |  |  |  |  |  | **1** |
| **Continuing Professional Development** | **X** |  |  |  |  |  | **1** |
| **Cooperation** | **X** |  |  |  |  |  | **1** |
| **Co-optation** | **X** |  |  |  |  |  | **1** |
| **Crossing the Quality Chasm** | **X** |  |  |  |  |  | **1** |
| **Data adj2 diffusing** |  |  |  |  |  | **X** | **1** |
| **Data adj2 diffusion** |  |  |  |  |  | **X** | **1** |
| **Data adj2 dissemination** |  |  |  |  |  | **X** | **1** |
| **Data adj2 exchange** |  |  |  |  |  | **X** | **1** |
| **Data adj2 linking** |  |  |  |  |  | **X** | **1** |
| **Data adj2 network?** |  |  |  |  |  | **X** | **1** |
| **Data adj2 partnership** |  |  |  |  |  | **X** | **1** |
| **Data adj2 shar$** |  |  |  |  |  | **X** | **1** |
| **Data adj2 transfer** |  |  |  |  |  | **X** | **1** |
| **Data adj2 translat$** |  |  |  |  |  | **X** | **1** |
| **Data broker** |  |  |  | **X** |  |  | **1** |
| **Data exchange** |  |  |  | **X** |  |  | **1** |
| **Data generation** |  |  |  | **X** |  |  | **1** |
| **Data mobili#ation** |  |  |  | **X** |  |  | **1** |
| **Data transfer** |  |  |  | **X** |  |  | **1** |
| **Data translation** |  |  |  | **X** |  |  | **1** |
| **Data uptake** |  |  |  | **X** |  |  | **1** |
| **Decision making** |  |  |  |  | **X** |  | **1** |
| **Disconfirmatory utili#ation** | **X** |  |  |  |  |  | **1** |
| **Educational$ influential$** | **X** |  |  |  |  |  | **1** |
| **Effectiveness research** | **X** |  |  |  |  |  | **1** |
| **end of grant knowledge translation** | **X** |  |  |  |  |  | **1** |
| **Evaluation research** | **X** |  |  |  |  |  | **1** |
| **evidence adj2 aware$** |  |  |  |  |  | **X** | **1** |
| **K* Term** | **KT Wiki** | **LaRocca** | **McKibbon** | **Mitton** | **Scott** | **Van Eerd** | **Count** |
| **evidence adj2 exchange** |  |  |  |  |  | **X** | **1** |
| **evidence adj2 impact** |  |  |  |  |  | **X** | **1** |
| **evidence adj2 link$** |  |  |  |  |  | **X** | **1** |
| **evidence adj2 network?** |  |  |  |  |  | **X** | **1** |
| **evidence adj2 partnership** |  |  |  |  |  | **X** | **1** |
| **evidence adj2 shar$** |  |  |  |  |  | **X** | **1** |
| **evidence adj2 transfer** |  |  |  |  |  | **X** | **1** |
| **evidence adj2 uptak$** |  |  |  |  |  | **X** | **1** |
| **Evidence based healthcare** | **X** |  |  |  |  |  | **1** |
| **Evidence based nursing** | **X** |  |  |  |  |  | **1** |
| **Evidence broker** |  |  |  | **X** |  |  | **1** |
| **Evidence exchange** |  |  |  | **X** |  |  | **1** |
| **Evidence generation** |  |  |  | **X** |  |  | **1** |
| **Evidence informed decision making** |  |  |  |  |  | **X** | **1** |
| **Evidence informed practi#e** | **X** |  |  |  |  |  | **1** |
| **Evidence mobili#ation** |  |  |  | **X** |  |  | **1** |
| **Evidence transfer** |  |  |  | **X** |  |  | **1** |
| **Evidence translation** |  |  |  | **X** |  |  | **1** |
| **Evidence uptake** |  |  |  | **X** |  |  | **1** |
| **Evidence-based decision making** |  |  |  |  |  | **X** | **1** |
| **External validation** | **X** |  |  |  |  |  | **1** |
| **Feedback** | **X** |  |  |  |  |  | **1** |
| **Gap Analysis** | **X** |  |  |  |  |  | **1** |
| **guideline adherence** |  |  |  |  |  | **X** | **1** |
| **Guideline Implementation** | **X** |  |  |  |  |  | **1** |
| **Guideline* adj3 diffusion** |  |  |  |  | **X** |  | **1** |
| **Guideline* adj3 implementation** |  |  |  |  | **X** |  | **1** |
| **Guideline* adj3 translation** |  |  |  |  | **X** |  | **1** |
| **K* Term** | **KT Wiki** | **LaRocca** | **McKibbon** | **Mitton** | **Scott** | **Van Eerd** | **Count** |
| **Guideline* adj3 utili#ation** |  |  |  |  | **X** |  | **1** |
| **Healthcare innovation** | **X** |  |  |  |  |  | **1** |
| **impact** | **X** |  |  |  |  |  | **1** |
| **Implementation adj2 program$** |  |  |  |  | **X** |  | **1** |
| **Implementation adj2 strateg$** |  |  |  |  | **X** |  | **1** |
| **improv$ adj3 knowledge** |  |  |  |  |  | **X** | **1** |
| **improv$ adj3 learning** |  |  |  |  |  | **X** | **1** |
| **improv$ adj3 practi#e?** |  |  |  |  |  | **X** | **1** |
| **Increase adj2 implementation** |  |  |  |  | **X** |  | **1** |
| **Industry liaison** |  |  |  |  |  | **X** | **1** |
| **information adj2 diffusing** |  |  |  |  |  | **X** | **1** |
| **information adj2 diffusion** |  |  |  |  |  | **X** | **1** |
| **information adj2 dissemination** |  |  |  |  |  | **X** | **1** |
| **information adj2 exchange** |  |  |  |  |  | **X** | **1** |
| **information adj2 link$** |  |  |  |  |  | **X** | **1** |
| **information adj2 network?** |  |  |  |  |  | **X** | **1** |
| **information adj2 partnership** |  |  |  |  |  | **X** | **1** |
| **information adj2 shar$** |  |  |  |  |  | **X** | **1** |
| **information adj2 spread$** |  |  |  |  |  | **X** | **1** |
| **information adj2 transfer** |  |  |  |  |  | **X** | **1** |
| **information adj2 translat$** |  |  |  |  |  | **X** | **1** |
| **Information broker** |  |  |  | **X** |  |  | **1** |
| **Information dissemination** |  |  |  |  |  | **X** | **1** |
| **Information exchange** |  |  |  | **X** |  |  | **1** |
| **Information generation** |  |  |  | **X** |  |  | **1** |
| **Information mobili#ation** |  |  |  | **X** |  |  | **1** |
| **information science** | **X** |  |  |  |  |  | **1** |
| **K* Term** | **KT Wiki** | **LaRocca** | **McKibbon** | **Mitton** | **Scott** | **Van Eerd** | **Count** |
| **Information transfer** |  |  |  | **X** |  |  | **1** |
| **Information translation** |  |  |  | **X** |  |  | **1** |
| **Information uptake** |  |  |  | **X** |  |  | **1** |
| **innovation adaptation** | **X** |  |  |  |  |  | **1** |
| **innovation adj2 adopt$** |  |  |  |  |  | **X** | **1** |
| **innovation adj2 implement$** |  |  |  |  |  | **X** | **1** |
| **Innovation adoption** | **X** |  |  |  |  |  | **1** |
| **innovation adoption and diffusion** | **X** |  |  |  |  |  | **1** |
| **innovation development process** | **X** |  |  |  |  |  | **1** |
| **innovations in health service delivery and organization** | **X** |  |  |  |  |  | **1** |
| **Integrated knowledge transfer** |  |  |  |  |  | **X** | **1** |
| **internal validation** | **X** |  |  |  |  |  | **1** |
| **Know-do gap** | **X** |  |  |  |  |  | **1** |
| **knowledge adj2 diffusing** |  |  |  |  |  | **X** | **1** |
| **knowledge adj2 exchange** |  |  |  |  |  | **X** | **1** |
| **knowledge adj2 implementation** |  |  |  |  | **X** |  | **1** |
| **knowledge adj2 linking** |  |  |  |  |  | **X** | **1** |
| **knowledge adj2 network?** |  |  |  |  |  | **X** | **1** |
| **knowledge adj2 partnership** |  |  |  |  |  | **X** | **1** |
| **knowledge adj2 shar$** |  |  |  |  |  | **X** | **1** |
| **knowledge adj2 translat$** |  |  |  |  |  | **X** | **1** |
| **Knowledge adoption** | **X** |  |  |  |  |  | **1** |
| **Knowledge communication** | **X** |  |  |  |  |  | **1** |
| **Knowledge creation** |  |  |  |  |  | **X** | **1** |
| **Knowledge cycle** | **X** |  |  |  |  |  | **1** |
| **K* Term** | **KT Wiki** | **LaRocca** | **McKibbon** | **Mitton** | **Scott** | **Van Eerd** | **Count** |
| **Knowledge development** |  |  |  |  |  | **X** | **1** |
| **Knowledge Development and Application** | **X** |  |  |  |  |  | **1** |
| **Knowledge diffusion** | **X** |  |  |  |  |  | **1** |
| **Knowledge dissemination** | **X** |  |  |  |  |  | **1** |
| **Knowledge exchange and uptake** |  |  |  |  |  | **X** | **1** |
| **Knowledge into use** | **X** |  |  |  |  |  | **1** |
| **Knowledge network?** |  |  |  |  |  | **X** | **1** |
| **Knowledge production** | **X** |  |  |  |  |  | **1** |
| **Knowledge production and utilization** | **X** |  |  |  |  |  | **1** |
| **Knowledge synthesis** | **X** |  |  |  |  |  | **1** |
| **Knowledge transformation** | **X** |  |  |  |  |  | **1** |
| **Knowledge utili#ation** | **X** |  |  |  |  |  | **1** |
| **link$ evidence** |  |  |  |  |  | **X** | **1** |
| **link$ practice** |  |  |  |  |  | **X** | **1** |
| **link$ research** |  |  |  |  |  | **X** | **1** |
| **link$ science** |  |  |  |  |  | **X** | **1** |
| **Mindlines** | **X** |  |  |  |  |  | **1** |
| **Motivation** |  |  |  |  | **X** |  | **1** |
| **Opinion leader** | **X** |  |  |  |  |  | **1** |
| **Participatory Research** |  |  |  |  |  | **X** | **1** |
| **Patient safety** | **X** |  |  |  |  |  | **1** |
| **policies adj2 develop$** |  |  |  |  |  | **X** | **1** |
| **policy and practice** |  |  |  |  |  | **X** | **1** |
| **Policy making** |  |  |  |  |  | **X** | **1** |
| **Popularization of research** | **X** |  |  |  |  |  | **1** |
| **practice adj2 chang$** |  |  |  |  |  | **X** | **1** |
| **Practice based evidence** |  |  |  |  |  | **X** | **1** |
| **Product adoption and utilization** | **X** |  |  |  |  |  | **1** |
| **K* Term** | **KT Wiki** | **LaRocca** | **McKibbon** | **Mitton** | **Scott** | **Van Eerd** | **Count** |
| **program$ adj3 adopt$** |  |  |  |  |  | **X** | **1** |
| **Quality assurance, Health Care** | **X** |  |  |  |  |  | **1** |
| **research adj2 "use"** |  |  |  |  |  | **X** | **1** |
| **research adj2 aware$** |  |  |  |  |  | **X** | **1** |
| **research adj2 diffusion** |  |  |  |  | **X** |  | **1** |
| **research adj2 disseminat$** |  |  |  |  |  | **X** | **1** |
| **research adj2 impact$** |  |  |  |  |  | **X** | **1** |
| **research adj2 transfer$** |  |  |  |  |  | **X** | **1** |
| **research adj2 uptak$** |  |  |  |  |  | **X** | **1** |
| **Research capacity** | **X** |  |  |  |  |  | **1** |
| **Research implementation** | **X** |  |  |  |  |  | **1** |
| **Research integration** | **X** |  |  |  |  |  | **1** |
| **Research Practice Gap** | **X** |  |  |  |  |  | **1** |
| **Research utili#ation** | **X** |  |  |  |  |  | **1** |
| **return on investment** |  |  |  |  |  | **X** | **1** |
| **Routinization** | **X** |  |  |  |  |  | **1** |
| **science adj2 aware$** |  |  |  |  |  | **X** | **1** |
| **science adj2 impact$** |  |  |  |  |  | **X** | **1** |
| **science adj2 implement$** |  |  |  |  |  | **X** | **1** |
| **science adj2 uptak$** |  |  |  |  |  | **X** | **1** |
| **science adj2 utilizat$** |  |  |  |  |  | **X** | **1** |
| **Science communication** | **X** |  |  |  |  |  | **1** |
| **Science evidence** |  |  |  |  |  | **X** | **1** |
| **Self Efficacy** |  |  |  |  | **X** |  | **1** |
| **Sociology of Knowledge** | **X** |  |  |  |  |  | **1** |
| **Spread** | **X** |  |  |  |  |  | **1** |
| **Sustainability** | **X** |  |  |  |  |  | **1** |
| **Technology transfer** |  |  |  |  |  | **X** | **1** |
| **K* Term** | **KT Wiki** | **LaRocca** | **McKibbon** | **Mitton** | **Scott** | **Van Eerd** | **Count** |
| **Third Mission** | **X** |  |  |  |  |  | **1** |
| **Transfer** | **X** |  |  |  |  |  | **1** |
| **Translating research into practice** | **X** |  |  |  |  |  | **1** |
| **Translation** | **X** |  |  |  |  |  | **1** |
| **Translational medicine** | **X** |  |  |  |  |  | **1** |
| **Translational research phase I** | **X** |  |  |  |  |  | **1** |
| **Translational research phase II** | **X** |  |  |  |  |  | **1** |
| **Translational research phase III** | **X** |  |  |  |  |  | **1** |
| **Translational science** | **X** |  |  |  |  |  | **1** |
| **Transmission of knowledge** | **X** |  |  |  |  |  | **1** |
| **TRIP** | **X** |  |  |  |  |  | **1** |
| **Turning research into practice** | **X** |  |  |  |  |  | **1** |
| **validation** | **X** |  |  |  |  |  | **1** |
