# Supplementary material for: Use of social network analysis methods to study professional advice and performance among healthcare providers: a systematic review
Source: Syst Rev. 2017 Oct 23;6:208. doi: 10.1186/s13643-017-0597-1 (PMC5651641; doi:10.1186/s13643-017-0597-1)
Supplement: Supplementary file 4 — MEDLINE search strategy. (DOCX 18 kb) [file 13643_2017_597_MOESM4_ESM.docx]

### Additional File 4: MEDLINE Search Strategy

Ovid Medline (R)

1990 to January week 2 2015

Searched on 21 January 2015

530 records were retrieved (after duplicates removed)

#### CONCEPT 1: Social Network Analysis

| Line | Search terms | | Article retrieved |
| --- | --- | --- | --- |
| 1 | ((social network$) adj3 (analy$ method or analy$ technique$ or analy$ or approach or data$ or diagram$ or examin$ or explor$ or framework$ or indicator$ or information or inquiry or interaction$ or investigat$ or intervention$ or map$ or measure$ or method$ or metric or model or pattern or perspective$ or questionnaire$ or software or structure$ or survey$ or technique$ or theor$ or tool$)).ti,ab. | 1627 | |
| 2 | Network intervention$. ti,ab. | 77 | |
| 3 | Exp Interprofessional Relations/ | 56072 | |
| 4 | (network or networks) .ti, ab | 214954 | |
| 5 | 3 and 4 | 1203 | |
| 6 | exp Sociometric techniques/ | 1062 | |
| 7 | (sociometr$ or sociogram$ or sociomap$ or network structure).ti,ab. | 2825 | |
| 8 | (opinion leader$ or change agent$ or champion$ or knowledge broker$).tw. | 4547 | |
| 9 | 5 and ( 6 or 7 or 8) | 34 | |
| 10 | (UCINET or NetDraw or KrackPlot or NetMiner or StOCNET or GRADAP or NEGOPY or FATCAT or MultiNet or Agna or Blue Spider or DyNet or MDLogix Solutions or Network Workbench or Pajek or Sentinel Visualizer or SocNetV or visone or CID-ABM or C-IKNOW or Commetrix or MetaSight or Referral Web or SONIVIS or CiteSpace or E-Net or Ego Net or VennMaker or Financial Network Analyzer or PGRAPH or PermNet or CFinder or KeyPlayer or KliqFinder or Network Genie or ONA surveys or NodeXL or MatMan or yFiles or LibSNA or NetworkX or UrlNet or igraph or latentnet or RSiena or statnet or tnet).tw. | 117 | |
| 11 | 1 or 2 or 9 or 10 | 1780 | |

#### CONCEPT 2: Diffusion of Innovations

| Line | Search terms | | Article retrieved |
| --- | --- | --- | --- |
| 12 | exp diffusion of innovation/ | 15787 | |
| 13 | Diffusion of innovation$.tw. | 447 | |
| 14 | ((diffusion or diffusivity) adj3 (characteristic$ or evidence or information or innovation or innovation adaptation or innovation adoption or knowledge or mapS or measurementS or modelS or pathway$ or pattern$ or process$ or propert$ or simulation$ or strateg$ or stud$ or system$ or technique$ or theor$ or theory model$)).ti,ab. | 16396 | |
| 15 | ((Innovation adj1(adopt$ or implementation or adaptation or development process or sharing or exchange)) or (Program adopt$ or (innovation$ in service delivery and organi#ation) or (innovation adoption and diffusion))) .ti,ab. | 253 | |
| 16 | Innovation adj2 (adopt$ or implement$) | 252 | |
| 17 | 12 or 13 or 14 or 15 or 16 | 32026 | |

#### CONCEPT 3: Knowledge Translation/Transfer

| Line | Search terms | | Article retrieved |
| --- | --- | --- | --- |
| 18 | exp organizational innovation/ or exp continuing education/ or exp policy/ or exp quality improvement/ or exp Total Quality Management/ or exp Knowledge management/ or exp evidence-based practice/ or exp evidence-based medicine/or exp Health Services Research/mt | 276358 | |
| 19 | (action research or adoption or applied dissemination or best practi?e or change or communit$ of practi?e or complex intervention or dissemination or effective dissemination or evaluation or guideline implementation or implementation or implementation research or implementation science or information or innovation or institutionali#ation or (linkage and exchange) or Participatory Action Research or policies or policy research or service innovation or translational research or “use” or utili#ation).ti,ab. | 1663002 | |
| 20 | (integrated knowledge translation or knowledge mobili#ation or knowledge to action or transmission of knowledge or knowledge network? or sharing knowledge).ti,ab. | 854 | |
| 21 | (knowledge adj2 (utili#? or uptake or transfer$ or disseminati$ or diffusi$ or translat$ or broker$ or exchange or generation or integration)).ti,ab. | 4387 | |
| 22 | ((Evidence adj2(exchange or transfer or uptake or diffus$ or implement$ or translat$ or utili#?)) or ((research adj2 (implement$ or transl$ or utili#?))).ti,ab. | 11872 | |
| 23 | ((information adj1(dissemination or exchange or transfer$ or transl$)) or ((data adj1(exchange or transfer or transl$))).ti,ab. | 6935 | |
| 24 | 18 or 19 or 20 or 21 or 22 or 23 | 1887679 | |

### Compilation

| Line | Search terms | | Article retrieved |
| --- | --- | --- | --- |
| 25 | 11 and 24 | 625 | |
| 26 | 11 and 17 | 39 | |
| 27 | 25 or 26 | 630 | |
| 28 | animals.sh | 5354095 | |
| 29 | 27 not 28 | 585 | |
| 30 | limit 29 to (English language and yr="1990 -Current") | 536 | |
|  | Duplicates | 6 | |
|  | Final | 530 | |

#### Key

/ = indexing term (MeSH heading)

exp = exploded MeSH heading

$ = truncation

? = optional character

.ti,ab. = terms in either title or abstract fields

adj3 = terms within three words of each other (any order)

.sh.= subject heading field

tw = text word
